# Influence of Target Width and Distance on Postural Adjustments in a Fencing Lunge

**DOI:** 10.5114/jhk/161572

**Published:** 2023-04-20

**Authors:** Anna Akbaş, Wojciech Marszałek, Anna Brachman, Grzegorz Juras

**Affiliations:** 1 Institute of Sport Sciences, Department of Human Motor Behavior, Academy of Physical Education, Katowice, Poland.

**Keywords:** Fitts’ law, speed-accuracy trade-off, anticipatory postural adjustments, early postural adjustments

## Abstract

The aim of this study was to examine whether target width and target distance influence the planning phase of a fencing lunge (early and anticipatory postural adjustments) as well as the execution phase of a fencing lunge. Eight elite female fencers participated in the study. The displacement of the center of foot pressure, muscle activity of the tibialis anterior, and kinematics of center of mass were recorded using force plates. The results show that target width and distance have no effect on early and anticipatory postural adjustments as well as the acceleration and velocity of the center of mass at the moment of foot-off. However, a greater target distance was associated with a greater max center of mass acceleration and velocity, and larger target width resulted in a greater max center of mass acceleration during lunging (p < 0.05). We suppose that the effect of task parameters on preparing a fencing lunge may be mitigated due to the specific technique adopted by expert fencers and the ballistic nature of a fencing lunge.

## Introduction

There is general agreement that aiming movements which require greater precision are performed slower than movements performed for larger targets. This simple assumption is based on the speed-accuracy trade-off, commonly referred to as Fitts’ law. According to Fitts (1954), the time required to hit the target is related to the movement distance and target width and follows the equation: MT = a + b × (log_2_2A/W), where MT is movement time, a and b empirical constants, A is movement amplitude, and W is target width. A and W can be used as parameters for controlling the difficulty of a motor task (Fitts, 1954). When Fitts’ index of difficulty (ID = log_2_2A/W) increases, the motor task constraints arise and the performance becomes more demanding. Therefore, the formula for Fitts’ law can also be written as MT = a + b × ID.

More recent findings regarding the speed-accuracy trade-off have shown that reproducible changes in action (movement time) under different task requirements (target size and distance) occur at the level of action planning (Bonnetblanc et al., 2004; Gutman et al., 1993). Therefore, the consequence of a change in task parameters can be observed in the mechanisms that precede the movement initiation, i.e., early and anticipatory postural adjustment (EPA and APA, respectively) (Bertucco et al., 2013; Duarte and Latash, 2007). In principle, APA aims to counterbalance the postural disturbance and maintain postural stability (Belenkiy et al., 1967; Massion, 1992). In some motor tasks, APA may also be associated with the stabilization of the given joint and reduce the redundant degrees of freedom (Wang et al., 2018). EPA, on the other hand, plays a critical role in creating adequate mechanical conditions for the whole body movement. Both EPA and APA involve postural muscle activation and result in a shift of the center of pressure (COP) before the movement onset, whereas EPA occurs earlier (up to 500 ms) than APA (up to 250) in relation to movement initiation (t0) (Krishnan et al., 2011).

The efficient control of body posture, as well as accurate pointing while maintaining an appropriate distance from the opponent, are crucial for successful participation in sports such as fencing. However, surprisingly limited action has been taken to employ Fitts’ law in understanding performance of fencers. Fencers utilize a very specific movement pattern to ensure sufficient accuracy and dynamics, i.e., a fencing lunge (Sinclair et al., 2013). The lunge begins with the acceleration of the armed arm followed by the explosive extension of the rear leg, and finishes when the point of the blade touches the target before the front leg strikes the floor (Czajkowski, 2005; Stewart and Kopetka, 2005). It is worth noting that the lunge is a combination of two rather independent movements combined into a sequence—arm pointing and lunging—which can be ascribed to the controlled and ballistic phase, respectively (Juras and Słomka, 2013; Yiou and Do, 2000). Previous research has reported that the fencing lunge is preceded by both postural phenomena, EPA and APA (Akbaş et al., 2021b). Lunge-specific EPA includes the early and relatively small backward shift of the COP, which was found to facilitate the rapid displacement of the entire body toward an opponent (Akbaş et al., 2021b). APA involves the onset of the bioelectrical activity of the tibialis anterior (TA) of the front lower limb, hence it participates in dorsiflexion and ankle joint stabilization (Akbaş et al., 2021b; Guilhem et al., 2014; Suchanowski et al., 2011).

Although there are many studies which have investigated the effect of the change in task parameters (W and A) in various motor tasks, it is still unknown whether the width of the target and the distance from the opponent may affect the mechanisms that prepare the fencer’s body posture for rapid lunging and influence the fencing lunge kinematics. Previous have studies shown that the way one prepares for a change in task parameters is task-specific. For example, in a quick arm pointing action, EPA onset did not depend on movement amplitude or task ID, however, EPA magnitude scaled with Fitts’ ID (Bertucco et al., 2013). In another arm pointing task, APA magnitude was found to decrease with a decrease in target width (Bonnetblanc et al., 2004). In a lower limb reaching task, APA magnitude (integrated muscle activity in a given period of time) decreased when the target size increased, while APA onset (time of muscle activation in relation to t0) occurred earlier with longer target distances (Bertucco and Cesari, 2010). According to Bertucco and Cesari (2010), APA onset and magnitude are independently organized and therefore, react differently to the change in A and W.

The influence of task parameters on movement planning has also been found in whole body movements. In a study by Juras et al. (2009), participants were instructed to jump to a target of different widths and distances. Authors observed that APA onset scaled with movement distance and showed close to significant scaling with target width. According to those authors, the ballistic nature of the task may have mitigated the scaling of APA onset with the target size because participants were not able to affect MT during the flight phase.

The ambiguous effect of W and A has also been found in movement kinematics. Specifically, in a study by Bertucco et al. (2013) changes in distance scaled with peak velocity and acceleration, while no effect of target width on kinematics was observed. In contrast, in the Bonnetblanc et al.'s (2004) study, peak movement velocity scaled proportionally with target width, while no effect of distance was reported. Finally, according to Bertucco and Cesari (2010), peak velocity showed the same trend as MT and was influenced by both target distance and target width.

In the present study, we focused on the speed-accuracy trade-off in a sport-specific task. In particular, we aimed to assess whether target width and target distance would influence fencing lunge kinematics and the mechanisms that preceded the movement execution, i.e., early and anticipatory postural adjustments. We hypothesized that target distance would have an effect on EPA, APA, and COM kinematics. More specifically, the longer the distance from the target, the earlier the COP displacement onset (EPA onset), longer COP amplitude (EPA amplitude), smaller magnitude of muscle activity (APA), and greater velocity and acceleration of COM. We also hypothesized that no effect of target width on EPA, APA, and COM kinematics would be found due to the ballistic nature of the fencing lunge.

## Methods

### Participants

The study included eight elite female epee fencers (mean ± SD; age 23.1 ± 4.2 years, body height 176.2 ± 10.8 cm, body mass 63.3 ± 8.7 kg, preferred en garde stance width 40.91 ± 5.92 cm), all members of the Polish National Team, including medalists from the most prestigious competitions such as the European and World Championships, World University Games, and Youth Olympic Games. All of the included fencers had competed at the international level during the year of the experiment. Participants provided their informed written consent for voluntary participation in the study. The study was conducted according to the Declaration of Helsinki and approved by the Ethics Committee of the Academy of Physical Education in Katowice (3/2014) before its initiation.

### Measures

Two force plates (AMTI, AccuGait, USA) were lined up along the anterior-posterior (AP) direction, both 50 cm x 50 cm. A 16-bit analogue data acquisition device was used to capture data concurrently from both force plates at a 100 Hz sampling frequency (Measurement Computing, USB-1616FS, USA). According to the manufacturer’s instructions, ground reaction forces and moments of forces were computed from analogue data. The resultant COP was calculated in the AP direction, taking into account the offset between the force plate centers, and filtered with a 7 Hz, fourth-order, low-pass Butterworth filter.

A wireless surface EMG system (Noraxon, Teleymo DTS Desk Receiver, United States) with a gain of 500, Common Mode Rejection Ratio (CMRR) greater than 100 dB, and resolution of 16 bits, was used to record the electrical muscle activity of the tibialis anterior (TA) of the front limb at a sampling rate of 1,500 Hz. Surface electrodes were located according to the recommendations of SENIAM. Band-pass filtration (10–500 Hz) was used in the signal post-processing. To construct a linear envelope, the signal was rectified and filtered using a low-pass-2nd-order Butterworth filter with a cut-off frequency of 10 Hz.

### Design and Procedures

**Figure 1:**
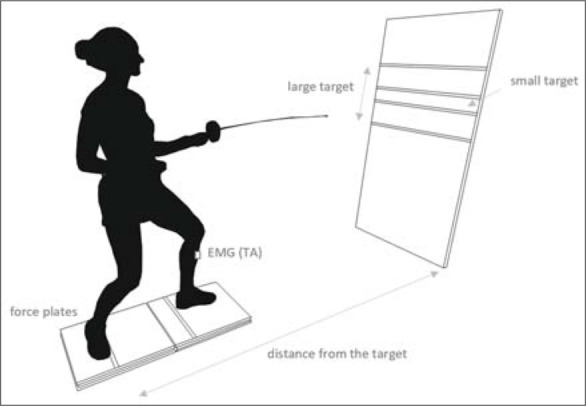
Experimental setup.

After a standard 10-min warm-up, participants were asked to perform several attacks with a lunge to become familiarized with the experimental set up. In order to avoid the effect of width of the initial en garde stance on APA and EPA throughout the different experimental conditions, distance between feet in the sagittal plane was standardized for each participant (Akbaş et al., 2021a). The stance width was calculated as the average distance between the heel of the front leg and the inner edge of the rear foot in the fencer’s natural en garde stance across three consecutive measurements. After that, participants were asked to position their en garde stance with one foot on each plate according to the lines marked on each plate (Figure 1). The instruction given to participants was typical of a Fitts task: perform an attack with a lunge as fast and as accurately as possible onto the target. Participants performed 10 trials of attacks with a lunge from three different distances from the target (short, medium, and long) and onto two different target sizes (small and large). The short distance corresponded to 145% of the participant’s body height, medium to 150%, and long to 155% (Akbaş et al., 2021b; Gutiérrez-Dávila et al., 2014). The size of the target was characterized by its height (10 cm—small, and 50 cm—large), while the width of the target was constant and limited only by the edges of the mattress. The trials were performed in a self-paced manner, meaning that participants could initiate the movement at will after adjusting and stabilizing the initial position on the force plates. Only those trials performed onto the target were analyzed. Therefore, participants were asked to perform the task for as long as it took them to reach 10 accurate trials. The time between consecutive trials was limited to the time required to return to the initial stance. The time between experimental conditions varied across participants, but was never shorter than 3 minutes to avoid the effects of fatigue. The order of the experimental conditions was randomized.

### Data Analysis

The Matlab (Math Works Inc., R2021b) software package was used to process the data. The onset of the lunge (t0) was defined as the time of front foot-off. This moment was determined based on the vertical ground reaction force (Fz). The following variables were calculated: EPA (COP amplitude, time to max amplitude, time from max amplitude to t0, and total duration of COP displacement), APA (magnitude of TA muscle activity), and COM kinematics (max acceleration, max velocity, time to achieve max acceleration and velocity, acceleration and velocity at t0).

The EPA variables were calculated from the resultant COP from both force plates in the AP direction. The onset of the COP displacement was determined based on the temporal velocity of the resultant COP signal (vCOP) in the AP direction in which positive and negative values corresponded to forward and backward movement, respectively. This point was determined as the last positive value before the vCOP became negative (start of the backward movement). Amplitude was defined as the maximal backward COP displacement which preceded t0. Time to max amplitude was defined as the time from the onset of backward COP displacement to the time in which this displacement was obtained. The total duration was the time from the onset of the backward COP displacement to t0. The time from max amplitude to t0 was the time interval between these two points.

To calculate the integrals of the muscle activity during the APA phase (∫EMG), the raw EMG data were first rectified and filtered using a Butterworth second order low-pass filter with a 100 Hz cutoff frequency. The ∫EMG was calculated in time interval of –250 ms to t0. The ∫EMG was corrected by the ∫EMG from the baseline which was calculated between –1250 and –1000 ms before t0. In order to normalize ∫EMG, the calculated values were divided by max ∫EMG obtained by a participant in the given condition.

COM acceleration and velocity were calculated from the force determined as the resultant ground reaction force in the AP direction from both force plates.

The index of difficulty (ID) was calculated according to the formula ID = log_2_2A/W, where A was movement amplitude and W was target width. Since the distance from the target was individualized to the participant’s height, the value of the ID differed between participants. Therefore, for each fencer, the ID was ranked from the easiest to the most difficult task (wide target: short, medium, long distance; narrow target: short, medium, long distance).

### Statistical Analysis

A 2 × 3 repeated measures ANOVA with factors ‘target width’ (2 levels: small, large) and ‘target distance’ (3 levels: short, medium, long) was utilized to assess the influence of target width and distance on EPA, APA, and COM kinematics. The Shapiro-Wilk test was used to verify the normality of the data distribution; all variables met this assumption. The Levene’s test was used to verify the homogeneity of the data variance of the samples; all data satisfied the assumption of variance homogeneity. The Mauchly’s sphericity test was used to validate assumptions for repeated measures ANOVAs; all variables met these assumptions. Significant effects were further explored using Tukey’s post-hoc analysis.

The Spearman’s rank test was used to evaluate the associations among the task ID and EPA, APA, and COM kinematics. The levels of correlation were considered weak (0–0.3), moderate (0.3–0.7), and high (0.7–1.0). Each test was performed in Statistica v.13.3 (TIBCO Software Inc.), and each test statistic was considered significant at the alpha level of 0.05. All effect sizes are reported as partial eta-squared (η2p).

## Results

The results presented as means and standard deviations are summarized in Table 1.

**Table 1:** Summary of the results of early postural adjustments, anticipatory postural adjustments, and COM kinematics.

Variable		Target size	Target distance		
***Early postural adjustments***					
	amplitude [cm]		short (mean ± SD)	medium (mean ± SD)	large (mean ± SD)
		narrow	0.470 ± 0.322	0.616 ± 0.313	0.381 ± 0.263
		large	0.495 ± 0.301	0.544 ± 0.236	0.432 ± 0.338
	time to max amplitude [s]				
		narrow	0.100 ± 0.019	0.106 ± 0.030	0.091 ± 0.016
		large	0.102 ± 0.014	0.103 ± 0.010	0.091 ± 0.029
	time from max amplitude to t_0_ [s]				
		narrow	0.308 ± 0.083	0.296 ± 0.089	0.321 ± 0.108
		large	0.299 ± 0.060	0.274 ± 0.053	0.314 ± 0.064
	total duration [s]				
		narrow	0.408 ± 0.084	0.402 ± 0.111	0.412 ± 0.110
		large	0.400 ± 0.062	0.376 ± 0.049	0.405 ± 0.051
***Anticipatory postural adjustments***					
	magnitude				
		narrow	0.738 ± 0.087	0.765 ± 0.110	0.703 ± 0.085
		large	0.749 ± 0.082	0.754 ± 0.051	0.714 ± 0.066
***COM kinematics***					
	max acceleration [m/s^2^]				
		narrow	4.704 ± 0.802	5.394 ± 0.845	5.535 ± 0.970
		large	5.265 ± 0.929	5.714 ± 0.809	5.935 ± 0.943
	time to max acceleration [s]				
		narrow	0.127 ± 0.078	0.159 ± 0.090	0.166 ± 0.078
		large	0.145 ± 0.067	0.175 ± 0.064	0.166 ± 0.075
	acceleration at t_0_ [m/s^2^]				
		narrow	3.543 ± 0.694	3.749 ± 0.964	3.722 ± 1.006
		large	3.520 ± 0.811	3.456 ± 0.721	3.501 ± 0.765
	max velocity [m/s]				
		narrow	1.466 ± 0.253	1.634 ± 0.268	1.702 ± 0.222
		large	1.503 ± 0.220	1.689 ± 0.221	1.750 ± 0.190
	time to max velocity [s]				
		narrow	0.296 ± 0.113	0.316 ± 0.095	0.316 ± 0.098
		large	0.298 ± 0.105	0.305 ± 0.064	0.301 ± 0.091
	velocity at t_0_ [m/s]				
		narrow	0.496 ± 0.257	0.496 ± 0.266	0.501 ± 0.256
		large	0.445 ± 0.194	0.426 ± 0.186	0.458 ± 0.167

### Early Postural Adjustments

No effects of target width, target distance, or interaction between target width and distance were found on early postural adjustments (*p* > 0.05). Detailed results for each variable, i.e., COP amplitude, time to max amplitude, time from max amplitude to t0, and total duration of EPA, are presented in Table 2. The task ID did not show any correlation with COP amplitude (r = –0.074), time to max amplitude (r = –0.130), time from max amplitude to t0 (r = 0.052), and total duration of EPA (r = –0.029) (*p* > 0.05).

**Table 2:** Outcomes of rm-ANOVAs examining the effect of target width (small, large) and target distance (short, medium, long) on early and anticipatory postural adjustments.

	Variable	Effect	F	df	*p*	η^2^p
***Early postural adjustments***						
	amplitude					
		width	0.000	1.7	0.994	0.000
		distance	2.423	2.14	0.125	0.257
		width x distance	0.320	2.14	0.731	0.044
	time to max amplitude					
		width	0.005	1.7	0.946	0.001
		distance	2.926	2.14	0.087	0.295
		width x distance	0.103	2.14	0.903	0.014
	time from max amplitude to t_0_					
		width	0.221	1.7	0.652	0.031
		distance	2.307	2.14	0.136	0.248
		width x distance	0.357	2.14	0.706	0.048
	total duration					
		width	0.180	1.7	0.684	0.025
		distance	1.325	2.14	0.297	0.159
		width x distance	0.571	2.14	0.577	0.075
***Anticipatory postural adjustments***						
	magnitude					
		width	0.048	1.7	0.834	0.054
		distance	1.687	2.14	0.221	0.295
		width x distance	0.206	2.14	0.816	0.076

### Anticipatory Postural Adjustments

No effects of target width, target distance, or interaction between target width and distance were found on anticipatory postural adjustments (*p* > 0.05). Detailed results for the magnitude of the TA bioelectrical activity of the front leg are presented in Table 2. The task ID did not show any correlation with the magnitude of TA activity (r = –0.074) (*p* > 0.05).

### Kinematics of COM

**Figure 2:**
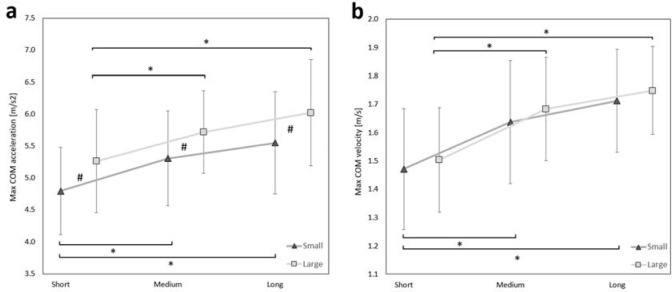
(a) Max COM acceleration and (b) max COM velocity for different target widths (small, large) and target distances (short, medium, long). The confidence intervals are presented as error bars. The significant post-hoc values between target width are marked as # and between distances as *.

The significant main effects of target width and target distance were found on max COM acceleration (Table 3). On average, fencers developed greater COM acceleration to the larger target when compared to the small one (5.67 vs. 5.22 m/s^2^). This effect was observed across all target distances: short (*p* = 0.002), medium, (*p* = 0.007), and long (*p* = 0.002) (Figure 2a#). Additionally, fencers accelerated faster from long and medium distances than from the short distance, while pointing to both small (short vs. medium *p* = 0.001, short vs. long *p* < 0.001 ) and large targets (short vs. medium *p* = 0.003, short vs. long *p* < 0.001) (Figure 2a*). The rm-ANOVA did not show an effect of interaction between target width and distance on max COM acceleration (*p* > 0.05).

**Table 3:** Outcomes of rm-ANOVAs examining the effect of the target width (small, large) and target distance (short, medium, long) on COM kinematics.

	Variable	Effect	F	df	*p*	η2p
***COM kinematics***						
	max acceleration					
		width	11.814	1.7	0.011*	0.628
		distance	31.237	2.14	<0.001*	0.817
		width x distance	0.128	2.14	0.881	0.018
	time to max acceleration					
		width	0.002	1.7	0.476	0.075
		distance	16.013	2.14	<0.001*	0.696
		width x distance	0.476	2.14	0.631	0.064
	acceleration at t_0_					
		width	0.254	1.7	0.630	0.035
		distance	0.502	2.14	0.616	0.067
		width x distance	0.059	2.14	0.943	0.008
	max velocity					
		width	1.697	1.7	0.234	0.195
		distance	59.082	2.14	<0.001*	0.894
		width x distance	0.060	2.14	0.942	0.009
	time to max velocity					
		width	0.405	1.7	0.545	0.055
		distance	1.289	2.14	0.306	0.156
		width x distance	0.285	2.14	0.756	0.039
	velocity at t_0_					
		width	0.555	1.7	0.481	0.073
		distance	0.704	2.14	0.511	0.091
		width x distance	0.138	2.14	0.872	0.019

* *statistically significant (p < 0.05)*

The significant main effect of target distance was found on max COM velocity (Table 3). The fencers developed greater max COM velocity in long and medium distances than in the short distance, for both small (short vs. medium *p* < 0.001, short vs. long *p* < 0.001) and large targets (short vs. medium *p* < 0.001, short vs. long *p* < 0.001) (Figure 2b*). The rm-ANOVA did not show an effect of target width and interaction on max COM velocity (*p* > 0.05) (Table 3).

The significant main effect of target distance was found on time to max COM acceleration (Table 2). The post-hoc analysis did not show any additional differences.

In addition, no effects of target width, distance, and interaction were found on COM acceleration and velocity at the moment of t0 and on time to max velocity (*p* > 0.05) (Table 3).

## Discussion

The aim of the present study was to investigate whether external performance conditions, such as target width and target distance, influenced postural control mechanisms, i.e., early and anticipatory postural adjustments (EPA and APA), and fencing lunge kinematics. We hypothesized that target distance would have an effect on all included variables. In particular, we hypothesized that the longer the distance, the earlier the onset of COP displacement (EPA onset), longer COP amplitude (EPA amplitude), smaller magnitude of muscle activity (APA), and greater velocity and acceleration of COM. We also hypothesized that no effect of target width on EPA, APA, and COM kinematics would be found in a fencing lunge because of its ballistic component.

The results did not support our first hypothesis that the distance would influence EPA and APA. In fact, neither target distance nor target width had an effect on EPA or APA. In previous studies, the scaling of feet-forward postural control mechanisms with task parameters was considered as evidence that the speed-accuracy trade-off takes place at the stage of movement planning (Bertucco and Cesari, 2010; Duarte and Latash, 2007; Gutman et al., 1993). Although the present results do not support this theory, it is possible that the effect of both target distance and width in a fencing lunge could be attenuated for two different reasons. First, the effectiveness of a lunge in elite fencers may not depend to a great extent on postural preparation. Previous studies have shown that elite fencers are characterized by the delayed onset of EPA and APA when compared to untrained controls, and that lunging is preceded by an extremely small COP amplitude (EPA) before the front foot take-off (Akbaş et al., 2021a, 2021b). Although this has not been empirically verified yet, we suppose that fencers may use a strategy based on the differences between the relative positions of COP and COM (Akbaş et al., 2021b). In particular, the backward COP displacement induces the forward-orientated acceleration of the fencer’s COM (Yiou and Do, 2001). In fact, at the moment of transitioning from double to single (rear) leg support, the gap between the position of the COP and the COM is created (Yiou et al., 2017). As a consequence, the fencer’s entire body falls towards the front foot and the fencer uses the force of gravity to accelerate the body. In such a situation, the role of postural preparation acting on the fencer is attenuated. The fact that no effect of target distance and width was found on EPA, APA, as well as COM acceleration and velocity at the moment of take-off (t_0_), may confirm the assumption that postural adjustments do not play a key role in propelling the body forward.

Second, the scaling of EPA and APA with target distance and width may be mitigated due to the ballistic nature of a fencing lunge (Juras et al., 2009; Juras and Słomka, 2013; Molina et al., 2019). According to Langendorfer et al. (2011), the ballistic motor skill is characterized by a multi- segment coordination pattern and optimal energy transfer through the body, resulting in high distal segment velocities and high-power output. According to Molina et al. (2019), reducing speed in a ballistic task may not lead to an increase in movement accuracy. Firstly, the formation of a movement pattern that supports effective energy transfer along the kinetic chain is hindered by a reduction in movement speed. Secondly, when both speed and accuracy are equally important, the goal of increasing accuracy may not be attained as a result of a drop in the overall speed of execution. To confirm these assumptions, several studies have shown that athletes are able to throw or shoot close to their maximum speed and still achieve a high level of accuracy (Afzal et al., 2020; García et al., 2013; Manaf et al., 2021; van den Tillaar, 2020). Moreover, in a study of Malina (1969), no difference in throwing accuracy was found in any of the three following groups: one promoting speed, one promoting accuracy, and both. However, speed was significantly greater in the group which emphasized speed.

Although there was no effect of the change in movement parameter on the phase of movement planning, the change in target distance and width influenced the movement execution phase. In particular, there was an effect of target width on max COM acceleration, as well as target distance on both max COM acceleration and velocity. Our results are in line with Bertucco et al. (2013), who proposed that a change in movement distance scaled close to linearly with the peak acceleration and peak velocity of the movement. However, it is worth mentioning that although max acceleration and velocity increased with the target distance, they were achieved at a similar time. Additionally, the fencers accelerated more slowly when they were moving towards a small target. According to Gutman et al. (1993), this acceleration constraints are of a mental nature and participants were simply scared to accelerate the body faster towards a very small target.

## Conclusions

In a fencing lunge, target distance and target width neither influence early and anticipatory postural adjustments nor COM acceleration and velocity at the moment of foot-off. However, in the movement execution phase, fencers accelerate faster and achieve greater COM velocities for longer distances, and they accelerate more slowly when aiming at a small target. We suppose that the effect of task parameters on fencing lunge preparation may be mitigated due to the specific technique adopted by expert fencers or due to the ballistic nature of a fencing lunge.

## Author Contributions

Conceptualization: A.A. and G.J.; methodology: A.A.; software: W.M. and A.B.; validation: W.M. and A.B.; formal analysis: A.A. and W.M.; investigation: A.A. and W.M.; resources: A.A.; data curation: A.A. and W.M.; writing—original draft preparation: A.A.; writing—review & editing: A.A., W.M. and A.B.; visualization: A.A. and W.M.; supervision: G.J.; project administration: G.J.; funding acquisition: G.J. All authors have read and agreed to the published version of the manuscript.

## ORCID iD

Anna Akbaş: 0000-0002-1244-1853

Wojciech Marszałek 0000-0003-3780-1090

Anna Brachman 0000-0002-3562-3131

Grzegorz Juras 0000-0001-8081-8854
